# Hsa-Mir-320c, Hsa-Mir-200c-3p, and Hsa-Mir-449c-5p as Potential Specific miRNA Biomarkers of COPD: A Pilot Study

**DOI:** 10.3390/pathophysiology29020013

**Published:** 2022-03-29

**Authors:** Noemi Cerón-Pisa, Amanda Iglesias, Hanaa Shafiek, Aina Martín-Medina, Margalida Esteva-Socias, Josep Muncunill, Aarne Fleischer, Javier Verdú, Borja G. Cosío, Jaume Sauleda

**Affiliations:** 1 Instituto de Investigación Sanitaria Illes Balears (IdISBa), 07120 Palma de Mallorca, Spain; ceronpisa.n@gmail.com (N.C.-P.); ainamartin89@gmail.com (A.M.-M.); josep.muncunill@ssib.es (J.M.); aarne.fleischer@ssib.es (A.F.); franciscoj.verdu@ssib.es (J.V.); borja.cosio@ssib.es (B.G.C.); jaume.sauleda@ssib.es (J.S.); 2 Centro de Investigación Biomédica en Red in Respiratory Diseases (CIBERES), 28029 Madrid, Spain; 3 Chest Diseases Department, Faculty of Medicine, Alexandria University, Alexandria 21526, Egypt; whitecoat.med@gmail.com; 4 Department of Molecular Biology, Wallenberg Centre for Molecular Medicine, Umea University, 90187 Umea, Sweden; margalida.esteva@gmail.com; 5 Respiratory Medicine, Hospital Universitario Son Espases, 07120 Palma de Mallorca, Spain

**Keywords:** lung disease, COPD, miRNAs, biomarker

## Abstract

Chronic obstructive pulmonary disease (COPD) is a chronic inflammatory disease commonly induced by cigarette smoke. The expression of miRNAs can be altered in patients with COPD and could be used as a biomarker. We aimed to identify a panel of miRNAs in bronchoalveolar lavage (BAL) to differentiate COPD patients from smokers and non-smokers with normal lung function. Accordingly, forty-five subjects classified as COPD, smokers, and non-smokers (*n* = 15 per group) underwent clinical, functional characterization and bronchoscopy with BAL. The mean age of the studied population was 61.61 ± 12.95 years, BMI 25.72 ± 3.82 Kg/m^2^, FEV1/FVC 68.37 ± 12.00%, and FEV1 80.07 ± 23.63% predicted. According to microarray analysis, three miRNAs of the most upregulated were chosen: miR-320c, miR-200c-3p, and miR-449c-5p. These miRNAs were validated by qPCR and were shown to be differently expressed in COPD patients. ROC analysis showed that these three miRNAs together had an area under the curve of 0.89 in differentiating COPD from controls. Moreover, in silico analysis of candidate miRNAs by DIANA-miRPath showed potential involvement in the EGFR and Hippo pathways. These results suggest a specific 3-miRNA signature that could be potentially used as a biomarker to distinguish COPD patients from smokers and non-smoker subjects.

## 1. Introduction

Chronic obstructive pulmonary disease (COPD) is currently the third leading cause of death worldwide and expected to increase in the near future [1]. Since more than 60% of patients are currently underdiagnosed, one of the major goals to improve survival is to detect those individuals that are in an early stage of the disease [2]. COPD is characterized by an abnormal inflammatory response of the lungs caused primarily by tobacco exposure. This response is considered abnormal since it is increased when compared to what occurs in smokers with normal lung function. Moreover, it further progresses in severity and persists even after smoking cessation [3]. However, only 20% of smokers develop COPD, suggesting that specific factors, such as genetic susceptibility or alterations in the epigenetic machinery, may be important in the development of the disease [4]. microRNAs (miRNAs) are a class of small, evolutionarily conserved, non-coding RNAs capable of regulating gene expression by different mechanisms [5,6]. Until now, miRNAs are known to play an important role in the regulation of various biological processes, such as cell differentiation and proliferation, although they are also involved in pathological processes; for instance, they can influence the progression of various types of human cancers by binding to the target sites of specific genes [6]. miRNAs fundamentally regulate these processes by acting as repressors of gene expression at the post-transcriptional level through the degradation of messenger RNA (mRNA) or the inhibition of its protein translation. Therefore, miRNAs have a great capacity to influence the expression of a large part of the genome and the intricate control system in which they are immersed, and their deregulation negatively affects the homeostasis of the organism [5]. They can be found in various biological samples, including bronchoalveolar lavage (BAL), which makes them interesting as biomarkers. In this regard, a panel of miRNAs have been related to inflammation in chronic lung diseases [7] and specifically to COPD and lung cancer (LC), suggesting that alterations in miRNAs may be a mechanism of COPD. Moreover, tobacco exposure could be the triggering factor for the miRNA down/upregulation [4,8], which suggests that microRNAs profiling could be an emerging tool to differentiate those patients with COPD from smokers that have not developed the disease.

We hypothesized, therefore, that changes in the level of certain miRNAs could be used as specific biomarkers in patients with COPD. Accordingly, in this study, we aimed to develop a panel of miRNAs in BAL that would allow us to detect and differentiate patients with COPD from the smokers and non-smokers. To this purpose, we performed a single-center, cross-sectional case-control in 45 subjects that were divided into COPD, smokers, and non-smokers with normal lung function to study the miRNA profile in BAL. Furthermore, we carried out an in silico analysis to study which pathological processes might be regulated by the candidate miRNAs that were found differentially expressed in the COPD group.

## 2. Materials and Methods

### 2.1. Study Design and Ethics

We present a single-center, cross-sectional case-control study that recruited patients at the Hospital Universitari Son Espases (Palma de Mallorca) who had clinical indications for bronchoscopy: mostly hemoptysis, lung nodule, or lung mass that later resulted negative for infection or lung cancer. The patients were then classified into groups based on the results of bronchoscopy, chest computed tomography (CT) (+/− fine needle aspiration), and spirometry. The project was approved by the Ethics Committee of the Balearic Islands in Spain.

### 2.2. Patients

Participants were selected from individuals referred to the pneumology outpatient clinic and bronchoscopy areas. To group the individuals according to their smoking status, we used the term “pack-year”, defined as the number of packs smoked per day multiplied by the number of years the person has smoked. The non-smoker group included patients with normal lung function and zero pack-years. The smokers with normal lung function and COPD patients with a cumulative smoking exposure of at least 10 pack-years were selected for the study. Participants were grouped by gender and age. At the time of recruitment, all COPD patients were stable, which is defined by the absence of exacerbations and stability in treatment for the last three months. COPD diagnosis and its severity were defined using post-bronchodilator spirometry indicators according to the Global Initiative in Obstructive Lung Diseases (GOLD) recommendations [3]. The exclusion criteria were other disorders or pathologies linked to inflammation: a history of asthma, atopy, allergic rhinitis, autoimmune diseases, renal disorders, other malignancies, lung cancer, infectious diseases, and/or using immunomodulatory drugs. Patients were further divided into three groups: COPD, smokers, and non-smokers with normal lung function. All the patients underwent fiber optic bronchoscopy under conscious sedation. Bronchoscopy was performed in the right or left lung for diagnosis purposes, and BAL was then obtained from the other part of the lung that remained free from the bronchoscopy procedure (either middle lobe or lingula) using standardized methods as described by ERS guideline [9]. The BAL was centrifuged at 1800 rpm for 8 min, and the supernatant was frozen at −80 °C. One sample was sent to the microbiology department to rule out active infection. The other sample was sent to the cytology area. We excluded lung cancer with a negative result in CT and in the cytological analysis.

### 2.3. Total RNA Isolation from BAL

Following the manufacturer’s recommendations to use the same starting volume of biofluid to ensure consistent RNAinput, total RNA, including the miRNAs fraction, were isolated from 200 μL of BAL with the miRNeasy advanced serum/plasma kit (Qiagen, Spain) according to the manufacturer’s protocol. A total of 10^8^ copies of UniSp6 RNA Spike-in control (Exiqon, Spain) were added to BAL before RNA isolation. UniSp6 RNA Spike-in control and the circulating miRNA miR-16-5p were used for monitoring both RNA isolation and cDNA synthesis procedures by quantitative PCR (qPCR) [10].

### 2.4. miRNA Expression Profiling in BAL in a Discovery Set of Patients by Microarary

A discovery set of samples were selected from patients for microarray analysis. Two groups were considered: COPD (*n* = 5) and smoker (*n* = 5). Both groups were matched by sex. Accurate miRNA isolation was previously confirmed by qPCR for UniSp6 RNA Spike-in control and for the endogenous miR-16-5p. Cq values were consistent amongst all 10 samples. We used 8 μL of total RNA for direct labelling with the FlashTagTM Biotin HSR Labeling Kit, and the labelled samples were hybridized at 48 °C and 60 rpm for 42 h to the GeneChip^®^ miRNA 4.0 Array (Applied Biosystems, Thermo fisher, Waltham, MA, USA). Array normalization was executed with the Transcriptome Analysis Software 4.0 (Applied Biosystems, Thermo fisher) following the robust multichip analysis (RMA) and the detected above background (DABG) algorithms. The eBayes method was used to identify the differentially expressed miRNAs amongst groups and were considered biologically significant if they displayed a fold change value (FC; <−1.5 or >1.5) and *p*-value < 0.05. DIANA-miRPath v3.0 was used for KEGG pathway enrichment based on predicted miRNA targets provided by the DIANA-microT-CDS algorithm [11]. We studied the expression of miRNAs selected according to the eBayes method in other published studies with the expression profile available in the Gene Expression Omnibus (GEO).

### 2.5. Real Time Quantitative PCR

Candidate miRNAs were validated in the complete cohort of 45 patients (COPD, smoker, and non-smoker groups, *n* = 15 each). We used the miRCURY LNA Universal RT microRNA PCR System (Exiqon, Qiagen, Spain) for the detection of miRNAs by quantitative real-time PCR (qPCR) using SYBR green. According to the instructions of the reverse transcription reagent, 2 µL of total RNA was reverse transcribed into cDNA with miRCURY LNA RT Kit (Exiqon, Qiagen, Spain) and incubated for 60 min at 42 °C, 5 min at 95 °C to heat inactivate the reverse transcriptase, and stored at 4 °C. cDNA was diluted 20× with nuclease free water. Subsequent real-time PCR reactions were performed in duplicate with the miRCURY LNA SYBR Green PCR Kit (Exiqon, Qiagen, Spain) with LNA-specific primers using a CFX96 system (Bio-Rad, Sapin). The thermocycling conditions were as follows: 95 °C for 2 min, followed by 44 cycles of denaturation at 95 °C for 10 s, and annealing/extension at 56 °C for 1 min. Product specificity was confirmed in initial experiments by melting curve analysis, and PCR efficiency was calculated for each LNA specific primer. The expression of the housekeeping gene (miR-16-5p) was used as an internal control to normalize the expression of each target gene. Relative quantification with the 2^−∆∆Cq^ method [12] was used to evaluate the relative expression of miRNAs of interest.

### 2.6. Statistical Analysis

All the data were presented as mean ± standard deviation (SD) or number (*n*) and percentage (%), as appropriate. Comparison between experimental groups was performed using analysis of variance (One-way ANOVA) followed by Bonferroni post hoc to compare different groups. A *p*-value of <0.05 was considered to represent a statistically significant difference. All statistical analysis was carried out using GraphPad InStat version 9.00 (GraphPad Software, Inc., La Jolla, CA, USA).

## 3. Results

### 3.1. Patient Characteristics by Group

Forty-five subjects were recruited and classified into three groups: COPD, smoker, and non-smoker with normal lung function (15 patients for each group). Twenty-one of them (46.67%) were men, and twenty-four (53.33%) were women. The mean age of the studied population was 61.61 ± 12.95 years, BMI 25.72 ± 3.82 Kg/m^2^, FEV1/FVC 68.37 ± 12.00%, and FEV1 80.07 ± 23.63% predicted. The FEV1/FVC % and the FEV1 % was significantly reduced in the COPD group compared to both smokers and non-smokers. According to the standard classification of the Global Initiative for Chronic Obstructive Lung Disease [13], patients were classified in GOLD 1 to 4. About 13.3% of the COPD patients were GOLD 1, and 66.7% were GOLD 2 (Table 1). Table 1 shows a summary of the baseline and clinical characteristics of the studied subjects.

### 3.2. BAL miRNA Profile in Patients

**Figure 1:**
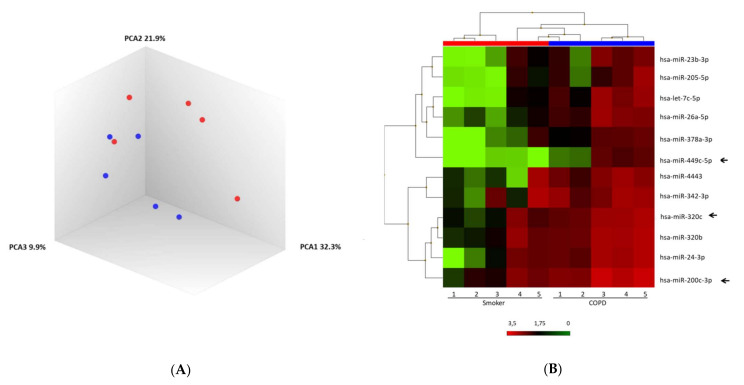
MiRNA microarray profiling of COPD and smoker. (**A**) Three-dimensional principal component analysis (PCA) plot showing the normalized signal values of ten expression profiles using BAL samples from chronic obstructive pulmonary disease (COPD) (blue) and smoker (red). (**B**) Heat map representation of an unsupervised hierarchical clustering of the ten most significant differentially expressed BAL-derived miRNAs in comparison between COPD and smokers. Arrows show miR-320c, miR-200c-3p, and miR-449c-5p as candidates.

A discovery set of 10 samples for the groups of COPD and smoker was pre-selected for microarray analysis. Principal component analysis of the normalized signal values showed that COPD and smoker samples appeared to be consistent among the individuals in each group, thus distributing the samples into two differentiated clusters (Figure 1A). Significant differences in miRNA expression were found between the two groups. Overall, 51 mature miRNAs were down-regulated, and 93 miRNAs were upregulated. A heatmap of unsupervised clustering analysis of all significant differentially expressed miRNAs related to COPD and smokers is shown in Figure 1B.

### 3.3. Validation of the Candidate miRNAs in the Entire Cohort by qPCR

**Figure 2:**
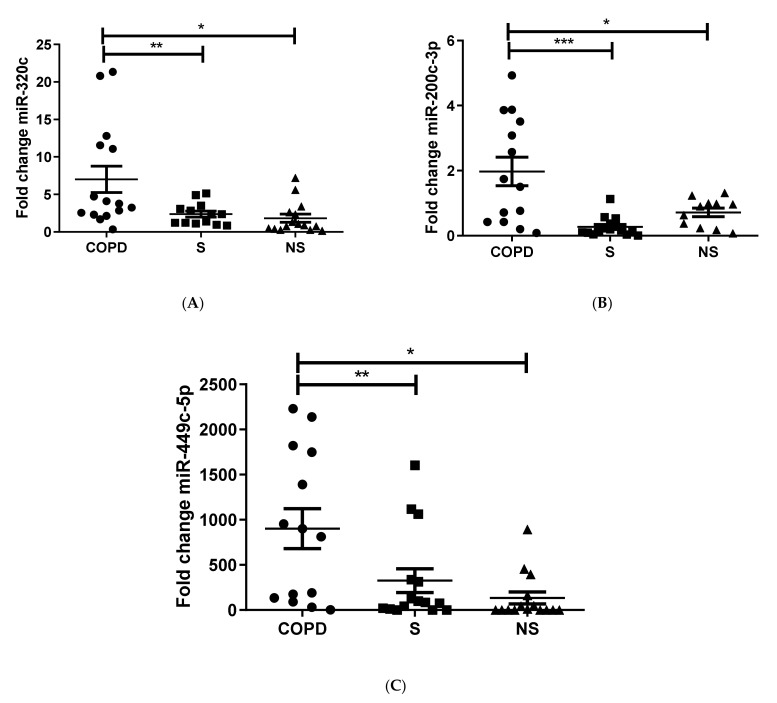
Validation miR-320c (**A**), miR-200c-3p (**B**), and miR-449c-5p (**C**). miRNA expression levels in BAL from the complete cohort (*n* = 15 per group). Results are represented as means ± SEM. One-Way ANOVA. * *p* < 0.05, ** *p* < 0.01, *** *p* < 0.001 compared to the COPD group. S, smoker; NS, non-smoker; COPD, chronic obstructive pulmonary disease.

Upon revision of the microarray results, miR-320c, miR-200c-3p, and miR-449c-5p were identified based on significance, previous studies, and standard deviation as possible candidates that could potentially differ in expression between COPD patients and smokers. Therefore, we proceeded to validate this 3-miRNAs signature in the complete sample of 45 patients by qPCR. We found that miR-320c was significantly upregulated in the group of COPD patients when compared to smokers (*p* = 0.03) and non-smokers (0.0025) (Figure 2A), miR-200c-3p was also significantly overexpressed in COPD versus smokers (*p* = 0.0218) and non-smokers (*p* = 0.0064) (Figure 2B), and miR-449c-5p was likewise significantly upregulated in COPD in comparison to smokers (*p* = 0.002) and non-smokers (*p* = 0.0112) (Figure 2C). In summary, all three miRNAs studied were significantly upregulated in the COPD in comparison to the other groups, therefore confirming the results seen in the microarray.

**Figure 3:**
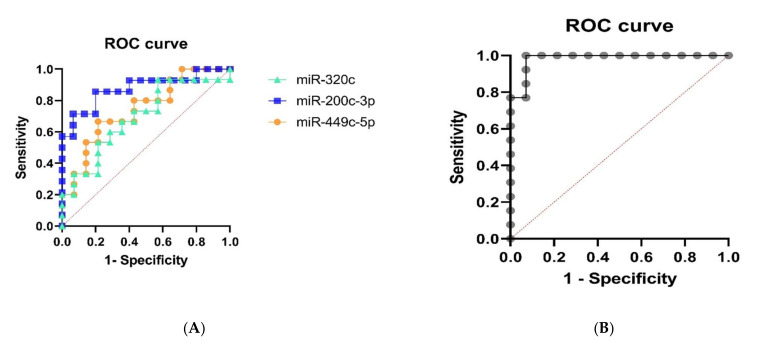
Receiver operating characteristic (ROC) curve for the individual mi-R-320c (AUC = 0.69), miR-200c-3p (AUC = 0.87), and miR-449c-5p (AUC = 0.74) (**A**) and for the logistic regression model (AUC = 0.89) (**B**).

To strengthen our results, we next performed ROC curves to see if the three miRNA candidates are capable of discriminating the COPD group from the smokers group. In general, miR-200c-3p and miR-449c-5p significantly (*p* < 0.05) discriminated COPD patients from the smoker group with an AUC of 0.87 and 0.74, respectively. On the other hand, miR-320c could not discriminate significantly (AUC of 0.69) (Figure 3A). A multivariate logistic regression analysis was further performed including the three miRNA in the model. The comparison of the expected and observed frequencies by the Log-likelihood relationship (*p*-value < 0.001) and by the ROC curve (AUC = 0.89, *p* < 0.001) indicated that the use of the three miRNAs together as biomarkers may be suitable for COPD (Figure 3B).

### 3.4. In Silico Analysis

**Figure 4:**
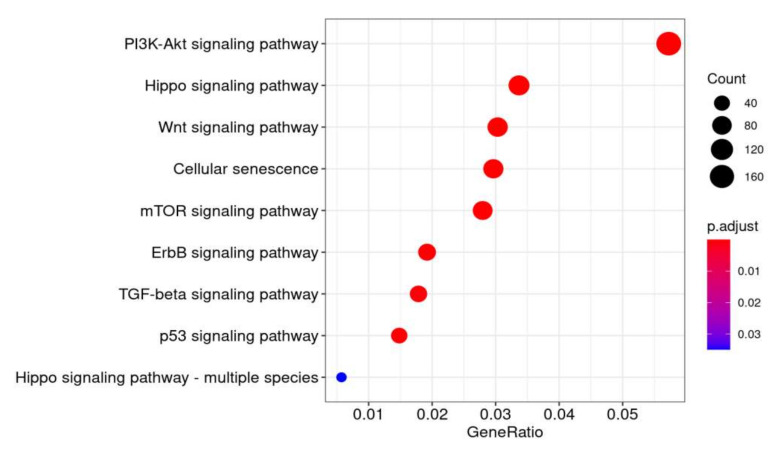
Functional analysis of common predicted gene targets for miR-320c, miR-200c-3p, and miR-449c-5p. DIANA-miRPath v3.0 was used for KEGG pathway enrichment based on predicted miRNA targets provided by the DIANA-microT-CDS algorithm. Gene ratios is the ratio of input genes that are annotated in a term. P-adjust is adjusted *p*-value. The method used for this correction was Benjamini–Hochberg (BH).

Finally, we carried out an in silico analysis to study the potential targets and pathways that are regulated by the three newly identified miRNAs. Firstly, the validated targets were identified in BAL from COPD, smokers, and non-smokers (1478 genes for miR-200c-3p, 221 genes for miR-449c-5p, and 754 for miR-320c). The functional enrichment analysis reported that the three miRNAs regulate different pathways that are involved in COPD processes, such as adherents junction, ErbB signaling pathway, Notch signaling pathway, and Wnt signaling pathway (miR-449c-5p); PI3K-Akt signaling pathway, p53 signaling pathway, tight junction, Hippo signaling pathway, ErbB signaling pathway, ubiquitin mediated proteolysis, TGF-beta signaling pathway, and Wnt signaling pathway (miR-200c-3p); and Hippo signaling pathway, cellular senescence, and mTOR signaling pathway (miR-320c) (Figure 4). This analysis suggests that the differential levels of miR-449c-5p, miR-320c, and miR-200-3p could not only serve as specific biomarkers for COPD patients but might also be useful as therapeutic targets since they potentially regulate various pathways involved in COPD pathogenesis. Further bioinformatic analysis was carried out to compare our results to those from other studies. Using the data available in the GEO repository, we identified three studies with similar characteristics to ours, which studied the expression profile of the same family related miRNAs to our selected miRNAs in COPD patients (GSE136390, GSE102915, GSE70080, GSE61741, GSE44531). miR-449 family miRNAs were found significantly overexpressed in COPD patients in one study (GSE61741: miR-449a, FC = 2.7, *p*-value = 0.0005). Contrary to our results, miR-200c was found to be downregulated in one case (GSE61741: FC = 0.59, *p*-value = 0.002). Moreover, miR-320 family was overexpressed (*p*-value = 0.018) in COPD patients (GSE70080: miR-320b, FC = 2.14). Therefore, this analysis allowed us to qualify two out of our three candidate miRNAs in other independent cohorts.

## 4. Discussion

COPD is characterized by an abnormal inflammatory response of the lungs caused mainly by tobacco. The decrease in expiratory airflow and the emphysema progresses along with severity even after the patient has stopped smoking [3]. The disease burden is one of the highest worldwide, and the elevated risk of underdiagnosis is one further challenge in COPD epidemiology [14]. For mild to moderate disease processes, more than 60% of patients are undiagnosed [2], and therefore, it is crucial to find novel biomarkers that allow for the detection of these patients. miRNAs are non-coding RNAs that regulate gene expression, and as their levels are often altered under disease conditions, increasing evidence point to them as good candidates for early biomarkers.

There is some evidence that in COPD, lung and blood cells express different amounts of miRNAs compared with healthy controls [15]. In fact, different expression profiles of miRNAs in blood cells, serum, and other corporal fluids can be used to discriminate between lung cancer patients and COPD patients [16,17]. COPD is associated with significant dysregulation of the immune system, which leads to a chronic inflammatory response. Several studies have reported the existence of differentially expressed miRNAs among individuals affected by respiratory pathologies, such as COPD and asthma, when compared with healthy individuals with a variable number of miRNAs associated depending on the type of sample analyzed [18]. Additionally, dysregulated miRNAs have been identified in lung tissue and other body fluids in COPD, including plasma, serum, sputum, bronchial lavage, saliva, and pleural fluid, compared to normal lung [10,19,20].

Here, we studied the miRNA profile in COPD, smokers, and non-smoker individuals to identify alterations that might allow to differentiate the COPD group from the rest. In an initial microarray analysis that included ten patients, we found that 51 miRNAs where downregulated, whereas 93 miRNAs appeared upregulated in the cohort of well-characterized COPD patients compared to smokers. We then decided to focus on those that exhibit a greater *p*-value and selected three candidate miRNAs, namely miR-200c-3p, miR-449c-5p, and miR-320c, for qPCR analysis including the complete cohort of 45 patients and confirmed that they were significantly upregulated in the COPD cohort compared to smokers and non-smokers. It must be mentioned that among all significantly differently expressed miRNAs detected by microarray analysis, the top ten candidates were all upregulated in COPD (including our three candidates). However, since miRNAs suppress gene expression, those that are significantly downregulated might also be interesting to study, as this results finally in upregulation of the target genes.

Despite tobacco being the most common cause of COPD, not all the smoker population will eventually develop the disease. Therefore, it might be possible that smoking is upregulating specific miRNAs in some of the smokers, thus conferring an additional risk for COPD. In fact, several authors have shown that tobacco smoke causes changes in gene expression of respiratory epithelium, which is linked to the development of respiratory diseases, such as COPD [4,19,21]. Conickx et al. observed that miR-200c-3p and miR-449c-5p were upregulated in murine lung tissue but not in BAL following 24 weeks of cigarette smoke exposure although they could not correlate the upregulation of these specific miRNAs in lung tissue from COPD. Here, we observed that both miR-200c-3p and miR-449c-5p (along with miR-320c) were indeed upregulated in BAL from patients with COPD compared with smokers and non-smokers, which might indicate that, in humans, an active secretion of these specific miRNAs takes place within the lung interstitium and alveolar space. Of note, the population used by the above-mentioned study included patients in COPD GOLD 2–3 (moderate to severe), whereas ours used patients mainly in GOLD 2 (moderate), which highlights the potential use of our three-miRNA profile as early biomarkers [4].

Apart from miR-200c-3p and miR-449c-5p, we also observed a significant increase in miR-320c in BAL from COPD patients compared to smokers and non-smokers. Similar to us, another study also found upregulated miR-320c in plasma from COPD patients and also in asthma-COPD overlap disease [22]. In addition, the miR-320 family has been associated with lung disease, especially due to its involvement in the regulation of AAT (alpha-1 antitrypsin) and the pathogenesis of AATD (AAT deficiency), which is a genetic risk factor associated with COPD [23,24]. Matamala et al. observed significantly higher levels of miR-320c in serum from healthy individuals with AAT deficiency compared to non-AAT, and importantly, miR-320c was also upregulated in individuals with obstructive lung disease (bronchiectasis, emphysema, chronic bronchitis, and asthma) compared to the healthy regardless of their AAT condition [25]. Of note, when they included an additional group of non-deficient COPD patients, the analysis revealed significantly higher miR-320c levels compared to healthy non-deficient subjects, in fact, similar to levels in AAT deficient individuals. In line with our results, they found no differences in miR-320c in smokers versus non-smokers, indicating that upregulation of miR-320c seems to be specific to obstructive lung disease [25]. In the current study, we propose that upregulation of miR-320c could not only serve as a biomarker for obstructive lung disease but could also be specific for COPD patients when it is found together with upregulated miR-200c-3p and miR-449c-5p. Both the above-mentioned studies observed a correlation between the upregulation of miRNAs and the inflammatory response, as Conickx et al. found that miR155 correlated with higher B-cell number [4], while Matamala et al. found that miR-320c increased in vitro after treatment with pro-inflammatory lipopolysaccharide [25]. Therefore, it would be interesting to further investigate whether our three-miRNA panel in BAL (miR-320c, miR-200c-3p, and miR-449c-5p) correlates with a pro-inflammatory response, for instance, by analyzing the levels of immune cells and pro-inflammatory cytokines in the BAL from the same COPD cohort.

In addition, let-7c has been reported to decrease with cigarette smoke exposure in a rat model of COPD [21], to be downregulated in COPD sputum, and to positively correlate with FEV1 [17]. We also observed downregulation of miR-let-7c in BAL from COPD subjects compared to non-smokers in the microarray analysis. However, we could not confirm this result when including the whole cohort in the qPCR analysis (data not shown).

Extracellular vesicles (EVs), such as exosomes, are small vesicles that can be secreted into the BAL and transport a variety of components, including miRNAs, and have been recently described to play a role in COPD [26]. Moreover, an emerging link between miRNA transfer by exosomes in COPD has been suggested [27,28], and cigarette smoke exposure has been found in vitro to modify several miRNAs secreted in exosomes from human primary small airway epithelial cells [29] and lung microvascular endothelial cells [30]. The study from Kaur et al. investigated the expression of miRNAs in exosomes from BAL of the same groups as in our study (COPD vs. smokers vs. non-smokers). They observed an upregulation of miR-320b and miR-22-3p, whereas miR-423-5p appears downregulated in COPD vs. non-smokers and miR-100-5p also downregulated in COPD vs. smokers. Like us, they observed no differences between smokers and non-smokers. Interestingly, they found a different miRNA profile (downregulation of miR-122-5p) when comparing lung tissue in the same groups [31]. The fact that the miRNA profiles studied in COPD widely differ depending on the biological sample of choice highlights the complexity of the miRNA role in disease which seems to depend, at least in part, on the specific cellular compartment and environment. Therefore, it would be important for future studies to perform analysis including both the EV as well as the EV-free fraction of the BAL.

The miRNAs that we observed differently expressed in COPD have also been found dysregulated in other inflammatory chronic lung diseases. For instance, whereas we observed upregulation of miR-320c in BAL from COPD subjects, downregulation in plasma from bronchial asthma patients has been reported by others [24,32]. In line with our results, Tang et al. found upregulated miR-200c in BAL cells from asthmatic patients [33], and another study based on meta-analysis of microarray datasets found upregulation of miR-449c (along with miR-34b) in asthma [34]. This overlap of miR-200c and miR-449c between COPD and asthma is probably due to their role in regulating key inflammatory responses in both diseases. In relation to other lung diseases, COPD is known to be a risk factor for developing lung cancer, especially for non-small cell lung cancer (NSCLC), and a common miRNA signature has been identified between both diseases by “miRNA set enrichment analysis” [35]. Of note, our three-miRNA signature (miR-200c-3p, miR-449c-5p, and miR-320c) is not present in that COPD-NSCLC panel, thus highlighting their potential use as biomarkers specifics of COPD only.

Deregulation of miRNAs translates into altered expression of molecules and subsequent alteration in the pathways involved. Differences in the expression of miRNAs involved in the remodeling of the extracellular matrix have been reported in several chronic lung diseases [36], and in particular, an increase has been shown in the levels of miR-15b in lung tissue from COPD patients, which correlates with disease severity [37], whereas a decrease was observed in miR-145-5p [23] and miR-181c [38]. The Hippo signaling pathway is a master regulator of ECM processes in lung development and disease [39]. Functional analysis of common predicted gene targets for miR-320c, miR-200c-3p, and miR-449c-5p performed in our cohort revealed that these three miRNAs could participate in the regulation of the “ErbB signaling pathway” and “Hippo signaling pathway” by targeting four (ERBB2, ERBB4, and ERBB3 and ERBB2IP) and six (MST1R, LATS1/2, SAV1, YAP, and TAZ) genes respectively involved in these pathways. Epidermal growth factor (EGF) receptors include HER1 (EGFR/ErbB1), HER2 (ErbB2), HER3 (ErbB3), and HER4 (ErbB4). Dysregulation of the EGFR pathway causes aberrant EGFR signaling, which is associated with the early-stage pathogenesis of respiratory diseases, such as cancer, lung fibrosis, COPD, asthma, and cystic fibrosis [40,41]. In addition, a recent study found that miR-101-3p.1, which is upregulated in COPD and especially in acute exacerbation COPD, activates the EGFR/PI3K/AKT pathway, thus facilitating disease progression [42]. On the other hand, the “Hippo signaling pathway”, where YAP and TAZ are downstream effectors, plays a crucial role in lung development and physiology. Dysregulation of the YAP/TAZ signaling pathway contributes to the development and progression of chronic lung diseases, such as lung cancer, pulmonary fibrosis, pulmonary hypertension, COPD, asthma, and lung infection [43]. Deficiency of TAZ in mice results in lung developmental abnormalities, which leads to an emphysematous lung phenotype that was frequently observed in patients with COPD [44]. Despite the good characterization of the involvement of Hippo pathways in COPD, studies about their (mis)regulation through miRNAs in disease are lacking. Other studies have also reported miRNA changes in COPD, for instance, an increase of some miRNAs was correlated with disease severity [45] and that modulation of SERPINE1 by miR-34c might be involved in disease progression [28]. We found no changes in miR-34c in our COPD cohort when compared to smokers and non-smokers. However, miR-320c and miR-449c (which we found significantly upregulated in COPD) have also been found to regulate SERPINE1 and to be higher in COPD lung tissue (miR-320c) [25] and in asthma (miR-449c) [34]. Although accurate functional studies should be performed to validate this in silico result, we suggest that upregulation of miR-320c, miR-200c-3p, and miR-449c-5p is potentially involved in dysregulation of the EGFR signaling pathway and Hippo modulators in COPD; therefore, targeting these miRNAs could be used as novel therapeutic approach for treating this respiratory disease.

BAL is a heterogeneous sample where several cell types can be found, including immune cells and epithelial cells; moreover, extracellular vesicles coming from any cell type present in the lung can potentially end up in the BAL as well [46]. Therefore, it is highly complex to elucidate which cell type is the origin of the miRNA deregulation. Nevertheless, our in silico data suggested involvement of miR-200c-3p, miR-449c-5p, and miR-320c in the regulation of the “Hippo signaling pathway”, whose deregulation in the alveolar epithelial cells (AECII) has been found to promote emphysema [47,48]. Thus, one could speculate that upregulation of these miRNAs might be happening in the AECII of the damaged epithelium from the COPD patient. On the other hand, peripheral blood mononuclear cells blood cells (PBMC) are key pro-inflammatory mediators of disease [49]. EGFR, a member of the “ErbB signaling pathway”, has also been found to mediate macrophage activation in the pathological context [50,51]. Macrophages are key immune effector cells in the pathogenesis of COPD, and BAL-macrophages from COPD patients have been reported to present a non-polarized phenotype that confers them a poor immune response [52,53]. As the “ErbB signaling pathway” was also detected in our in silico analysis, the three miRNAs candidates might be implicated in the alteration of the EGFR role in macrophage function in COPD. Future studies must be carried in order to better elucidate the miRNA role in specific cell types involved in COPD pathogenesis.

The size of our cohort was limited since this was a pilot study that aimed to give some preliminary insights in the miRNA profiling of BAL from COPD patients. To increase the reliability of our study, we conducted additional bioinformatics analysis in other similar studies, which confirmed upregulation of two out of our three candidate miRNAs (miR-449 and miR-320) in COPD. Nevertheless, the use of a larger sample size, especially in the discovery phase, will provide more result accuracy.

The limited size did not allow us to correlate the expression of our miRNAs with disease severity; however, we could evaluate the diagnostic potential of our three miRNA candidates by plotting ROC curves and found that miR-200c-3p and miR-449c-5p were both capable of discriminating COPD from smokers. Moreover, greater sensibility was reported when analyzing the three miRNAs, namely miR-200c-3p, miR-449c-5p, and miR-320c, as a group (AUC = 0.89, *p* < 0.001). In this regard, the study from Bersimbaev et al. reported a ROC curve of COPD patients for plasma levels of miR-320c with an AUC of 0.855. Although the values are closed to ours, we could not find a significance for miR-320c upon ROC analysis, which might be influenced by the limited size of our cohort and by the fact that Bersimbaev et al. used plasma samples instead of BAL. [22].

This study has analyzed the miRNA profile in BAL from COPD, smokers, and non-smoker individuals; however, miRNAs can also be extracted from liquid biopsies, such as blood collection. In fact, dysregulated miRNAs have been identified in lung tissue and other body fluids in COPD, including plasma, serum, sputum, bronchial lavage, saliva, and pleural fluid, compared to normal lung [10,19,20]. Even though bronchoscopy is an invasive technique, we chose BAL to identify key miRNAs modifications, as this sample type more closely reflects the physiological processes. Consequently, it would be important to validate these findings in less invasive materials, such as sputum or serum, in order to apply it to routine clinical practice. It is important to consider that the miRNA profile obtained may differ depending on the collection sample even when coming from a same individual. This fact is reflected in previous studies, such as that of Conickx et al., which included lung tissue and BAL [4], and highlights the importance to use the right panel of miRNAs for the right sample. Therefore, it would be interesting to extend this study by including biological samples other than BAL, such as serum or sputum.

Some of our findings slightly differ from previous studies; however, it needs to be considered that others have mostly used lung tissue to study the miRNA expression profile in COPD, whereas we used BAL, which contains material from a heterogeneous cell population. On the other hand, identifying new miRNA profiles in the early stages of disease that are specific for COPD in comparison to smokers could be used as a potential tool to predict which smokers could potentially develop COPD, thus facilitating the early treatment and monitoring of disease.

## 5. Conclusions

This pilot study included a cohort of subjects classified in COPD, smokers, and non-smokers that were subjected to miRNA profiling in BAL samples. Despite the limited size, our results suggest a three-miRNA upregulation profile of miR-320c, miR-200c-3p, and miR-449c-5p in BAL for the first time, which appears to predict the development of COPD in smokers. Consequently, the joint upregulation in miR-320c, miR-200c-3p, and miR-449c-5p levels could serve as potential BAL biomarker to better discriminate COPD from smoker and non-smoker subjects, but these results need to be validated in a new, independent cohort. Moreover, in silico analysis found that these three miRNAs are potentially involved in the regulation of EGFR and Hippo pathways, which are linked to COPD progression. Even though these findings need to be validated in a larger cohort, they are encouraging and open potential new avenues for the tailored treatment of COPD based on miRNA profiling.

## Figures and Tables

**Table 1:** Characteristics of the study population.

	COPD	Smoker	Non-Smoker
Group	1 (*n* = 15)	2 (*n* = 15)	3 (*n* = 15)
Age (years)	67.07 ± 12.24	55.66 ± 12.41	62.46 ± 12.38
Gender (M/F; *n*) (%)	8 (53.33)/7 (46.66)	10 (66.67)/5 (33.33) †	3 (20)/12 (80)
BMI (kg/m2)	26.12 ± 4.07	26.10 ± 3.10	24.58 ± 4.57
Pack-years	51.61 ± 42.16 †	36.66 ± 27.38†	0 ± 0
Smoking status			
Current smoker	7 (46.60%)	4 (26.60 %)	0 (0%)
Former smoker	8 (53.30%)	11 (73.30%)	0 (0%)
Non-smoker	0 (0%)	0 (0%)	15 (100%)
FEV1/FVC (%)	57.26 ± 8.78 *†	76.90 ± 4.63	76.83 ± 6.36
FEV1 (% Ref)	61.23 ± 18.17 *†	94.81 ± 17.10	97.00 ± 10.86
COPD Stage (%)			
GOLD 1	2 (13.30%)		
GOLD 2	10 (66.67%)		
GOLD 3	3 (20%)		
GOLD 4	0 (0%)		

Data represents mean and SD. COPD, chronic obstructive pulmonary disease. COPD stage used GOLD standard [3]. * *p* < 0.05 compared to Smoker. † *p* < 0.05 compared to non-smoker.

## Data Availability

Not applicable.
